# Pharmacogenetics of thiopurines

**DOI:** 10.20517/cdr.2019.004

**Published:** 2019-06-19

**Authors:** Raffaella Franca, Giulia Zudeh, Sofia Pagarin, Marco Rabusin, Marianna Lucafò, Gabriele Stocco, Giuliana Decorti

**Affiliations:** ^1^Department of Medical, Surgical and Health Sciences, University of Trieste, Trieste 34127, Italy.; ^2^PhD Course in Reproductive and Developmental Sciences, University of Trieste, Trieste 34127, Italy.; ^3^Department of Life Sciences, University of Trieste, Trieste 34127, Italy.; ^4^Institute for Maternal and Child Health I.R.C.C.S. Burlo Garofolo, Trieste 34127, Italy.; ^5^Experimental and Clinical Pharmacology Unit, Centro di riferimento oncologico, I.R.C.C.S., Aviano 33081, Italy.

**Keywords:** Thiopurines, acute lymphoblastic leukemia, therapy personalization, thiopurine methyltransferase, *NUDT15*, *PACSIN2*, inosine triphosphate pyrophosphatase, pharmacogenetics clinical implementation

## Abstract

Polychemotherapeutic protocols for the treatment of pediatric acute lymphoblastic leukemia (ALL) always include thiopurines. Specific approaches vary in terms of drugs, dosages and combinations. Such therapeutic schemes, including risk-adapted intensity, have been extremely successful for children with ALL who have reached an outstanding 5-year survival of greater than 90% in developed countries. Innovative drugs such as the proteasome inhibitor bortezomib and the bi-specific T cell engager blinatumomab are available to further improve therapeutic outcomes. Nevertheless, daily oral thiopurines remain the backbone maintenance or continuation therapy. Pharmacogenetics allows the personalization of thiopurine therapy in pediatric ALL and clinical guidelines to tailor therapy on the basis of genetic variants in *TPMT* and *NUDT15* genes are already available. Other genes of interest, such as *ITPA* and *PACSIN2*, have been implicated in interindividual variability in thiopurines efficacy and adverse effects and need additional research to be implemented in clinical protocols. In this review we will discuss current literature and clinical guidelines available to implement pharmacogenetics for tailoring therapy with thiopurines in pediatric ALL.

## Introduction

**Figure 1:**
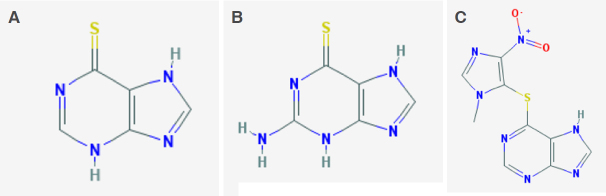
Thiopurines chemical structure. A: thioguanine; B: mercaptopurine; C: azathioprine

Thiopurines such as mercaptopurine and thioguanine (MP and TG, Figure 1A and B respectively) are lympholytic drugs used in all phases of therapy for acute lymphoblastic leukemia (ALL), with MP being part of the mainstay maintenance therapy^[1]^; instead, the MP prodrug azathioprine (AZA, Figure 1C) is employed in non-malignant conditions such as inflammatory bowel disease (IBD), including Crohn’s disease (CD) and ulcerative colitis (UC) during the maintenance phase of treatment^[2]^.

**Figure 2:**
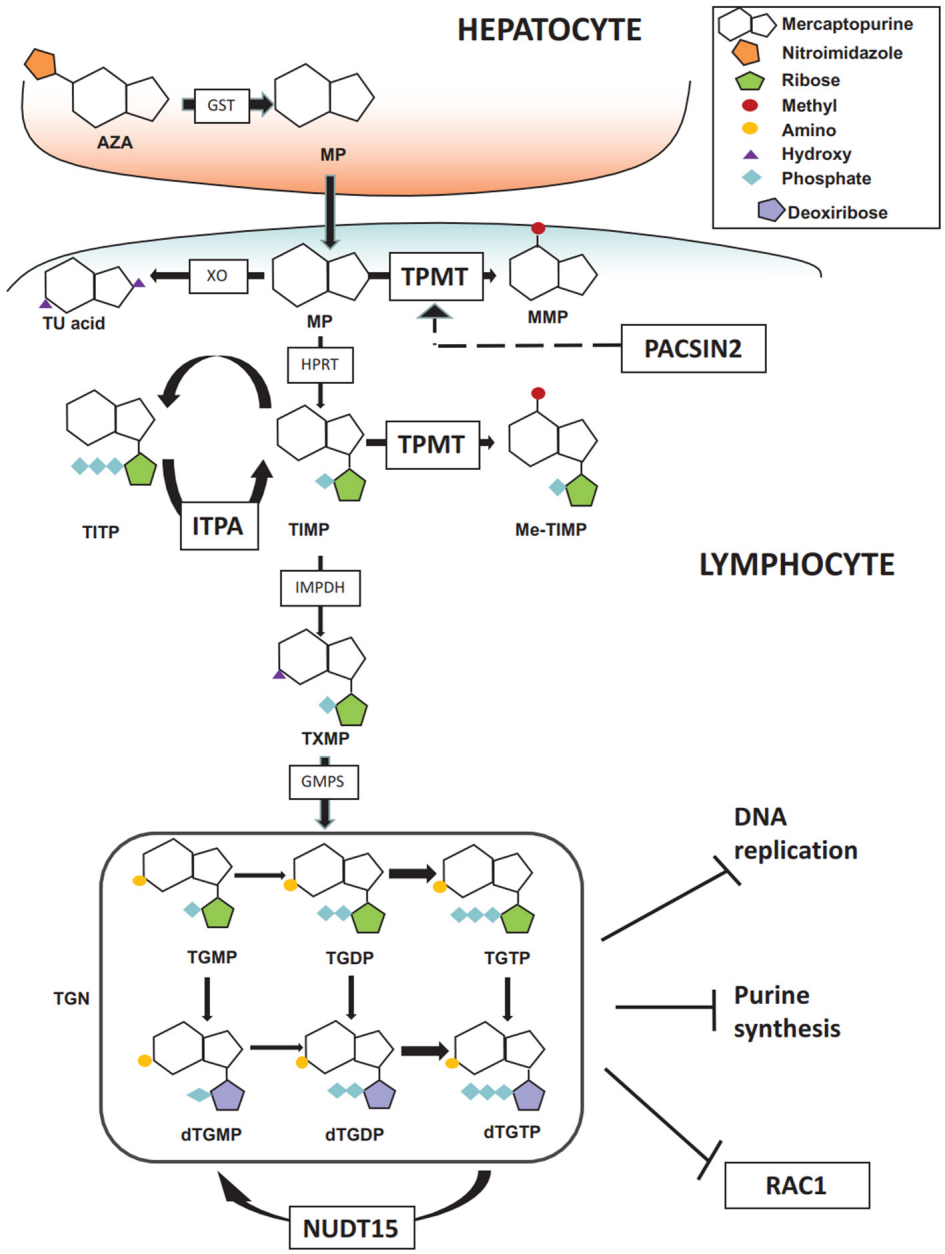
Thiopurine pathway. MP: mercaptopurine; MMP: methyl-mercaptopurine; TIMP: thioinosine monophosphate; Me-TIMP: methyl-thioinosine monophosphate; TGDP: thioguanine diphosphate; TGMP: thioguanine monophosphate; TGTP: thioguanine triphosphate; TITP: thioinosinetriphosphate; Me-TITP: methylthioinosinetriphosphate; TGN: thioguanine nucleotide; TU acid: thiouric acid; TXMP: thioxantine monophosphate; AZA: azathioprine; IMPDH: Inosine-5′-monophosphate dehydrogenase; ITPA: inosine triphosphate pyrophosphatase; GMPS: GMP synthase; GST: glutathione-transferase; NUDT15: nucleotide triphosphate diphosphatase gene; PACSIN2: Protein kinase C and casein kinase substrate in neurons protein 2; TPMT: thiopurine methyltransferase; XO: xanthine oxidase. Hatched arrow indicates the infleunce of *PACSIN2* on *TPMT* gene expression and TPMT activity

Thiopurines are antimetabolites similar in structure to purines: in particular MP is an analogue of hypoxanthine and TG of guanine. In cells, thiopurines undergo complex anabolic and catabolic processes. The anabolic pathway produces thionucleotides (TGN), including thioguanosine mono-, di-, tri-phosphate (tGMP, tGDP, tGTP) and deoxythioguanosine mono-, di-, tri-phosphate (tdGMP, tdGDP, tdGTP), associated with therapeutic efficacy. The conversion of MP to tdGMP/tGMP involves the consecutive action of the enzymes of salvage pathway of nucleotides biosynthesis, in particular hypoxanthine phosphoribosyltransferase 1 (HPRT1), inositol monophosphate dehydrogenase (IMPDH) and guanosine monophosphate synthetase (GMPS), whereas TG is directly converted by HPRT1 in a single step reaction [Figure 2]. The resulting tdGTP/tGTP thionucleotides antagonize the incorporation of canonical dGTP and GTP into DNA and RNA, thus impairing DNA and RNA polymerases and subsequently inducing cell-cycle arrest and apoptosis because of altered DNA, RNA and protein synthesis^[3]^. Furthermore, the cytotoxic action of these drugs in lymphoid cells is implemented by additional mechanisms of action such as the inhibition of the purine *de novo* synthesis pathway and tGTP-mediated inhibition of Ras-related C3 botulinum toxin substrate 1 (Rac-1), a GTPase of the Rho family^[3]^. Catabolic pathways of thiopurines are mediated by the enzymes xanthine oxidase (XO) and thiopurine methyltransferase (TPMT). The extensive first-pass metabolism of the drug by XO in the liver and intestinal mucosa is responsible for the low bioavailability of oral MP (less than 20%) and generates the main inactive metabolite, 6-thiouric acid, excreted in the urine^[4]^. Indeed, it is known that allopurinol, a structural isomer of hypoxanthine and a XO inhibitor, influences thiopurine pharmacokinetics and promotes TGN production^[4]^. MP and TG are also converted into methylmercaptopurine (MMP) derivatives by TPMT. This reaction can occur also in cells different from hepatocytes, since TPMT is ubiquitously expressed. MMP is not converted to nucleotides, as it is a poor HPRT substrate and has no antileukemic activity^[5]^. The synthesis of MMP therefore is in competition with the anabolic pathway of thiopurines. TPMT catalyses the S-methylation also of intermediate thionucleotides leading to TGN, producing secondary methylated nucleotides (MMPN), with potential cytotoxic activity through the inhibition of *de novo* purine synthesis. The balance between TGN and MMPN has been related to thiopurines response and cytotoxicity^[6]^. In IBD, TGN concentrations between 230 and 450 pmol/8 × 10^8^ red blood cells (RBC) are associated with the therapeutic index and clinical efficacy, while higher TGN concentrations have been related to myelosuppression and other severe complications such as infections^[7]^; concentrations of MMPN above 5,700 pmol/8 × 10^8^ RBC have been associated with hepatotoxicity^[8]^. To authors’ knowledge, there is no general consensus for the range of TGN/MMPN plasma concentrations that should be achieved for drug efficacy and toxicity in ALL: some clinical protocols suggest MP dose reduction with TGN concentration above 1000 pmol/8 × 10^8^ RBC in order to avoid excessive toxicity^[9]^. Thiopurine active metabolites persist in the blood for a long time in contrast to MP plasma concentration that rapidly declines after oral administration (MP serum peak is achieved within 2 h, half-life ranges from 20 to 120 min according to patients age and drug formulation): TGN are detected in RBC by the 3rd day of treatment and reach the steady state around the 3rd week; in IBD patients, clinical response to thiopurines is further delayed and appears between weeks 8 and 17 of therapy making these drugs unsuitable to achieve early disease remission but useful in long-term maintenance treatments^[10]^. TGN are routinely detected in RBC rather than in leukocytes, although RBC lack IMPDH and have therefore a thiopurine metabolism different from with that of principal target cells of these drugs. However, erythrocyte TGN levels have been found to correlate to leukocyte TGN levels in pediatric patients^[11,12]^.

In ALL, thiopurines are always used in the context of a polychemotherapeutic protocol that comprises glucocorticoids, vincristine, asparaginase, cytarabine, anthracyclines, cyclophosphamide and tyrosine-kinase inhibitors such as imatinib; in particular, MP is used in combination with methotrexate^[13-15]^. Specific ALL polychemotherapeutic approaches differ across worldwide protocols in terms of drugs, dosages and combinations; however, they consistently share the therapeutic scheme with an initial remission-induction phase to eradicate the burden of tumoral cells and restore normal hematopoiesis, followed by a remission-consolidation therapy and then by a long-term maintenance regimen (up to 24 months after diagnosis) to eliminate minimal residual leukemic cells. Such therapeutic scheme, combined with a risk-adapted intensive polychemotherapy, has been extremely successful for children with ALL who have reached an outstanding 5-year survival greater than 90% in developed countries. Two of the currently running main clinical trials in childhood ALL, the European AIEOP-BFM (Associazione Italiana di Ematologia ed Oncologia Pediatrica-Berlin-Frankfurt-Munster) 2017 protocol and the Total Therapy XVII (TOTAL XVII) used at the St. Jude Children’s Research Hospital (SJCRH) in Memphis, USA (ClinicalTrials.gov identifiers: NCT03643276 and NCT03117751, respectively http://clinicaltrials.gov) are aimed at further improving cure rate and quality of life of patients by exploring the effect of innovative drugs such as the proteasome inhibitor bortezomib and the bi-specific T cell engager blinatumomab. Nevertheless, thiopurines continue to be of interest: indeed, being administered daily throughout the duration of the entire therapy, although introduced 4/5 weeks after diagnosis and therapy start (i.e., after the induction phase), they remain the backbone of the polychemotherapy in both clinical trials. A detailed assessment of thiopurine metabolism and, in particular, of MP tolerance, toxicity and treatment outcome is one of the explorative objectives of the SJCRH TOTAL XVII protocol.

The most common drug related adverse effect of thiopurines is bone marrow suppression: leukopenia is a well-recognized effect occurring in more than 50% of ALL patients and 15%-20% in IBD patients^[16]^, leading to dose reductions or therapy interruption, with consequent increase of disease recurrence and infection rate. Gastrointestinal complication are also very common in terms of anorexia, diarrhea, nausea, vomiting and stomatitis; the use of thiopurines may be limited also by the development of pancreatitis, although this complication is very rare in ALL patients (< 5%)^[17]^. Among other drug related adverse effects, there are hepatic toxicity and flu-like symptoms.

## Pharmacogenetics

Pharmacogenetics of thiopurines in pediatric ALL is very well studied. Table 1 summarizes the genetic factors involved in thiopurine toxicities and further discussed in this review: *TPMT* variants have the major established role in clinics; clinical guidelines are now also available for *NUDT15* polymorphisms, whereas the pharmacogenetic contributions of other mentioned genes are either smaller or not yet steadily confirmed and therefore require further studies for their clinical implementation.

**Table 1:** Putative pharmacogenetic indicator for thiopurines

Gene	Protein function	Genetic variants	Influence on mercaptopurine response in patients	Ref.
*TPMT*	S-methylation*	rs1800462 (*2, 238C>G, pAla80Pro) rs1800460 (*3B, 460G>A, p.Ala154Thr) rs1142345 (*3C, 719A>G, p.Tyr240Cys)	Severe leukopenia and hematological toxicity in carriers of the variant alleles	[36]
*NUDT15*	Diphosphatase, catalyses the hydrolysis of nucleoside triphosphates and their oxidized forms	rs116855232 (415C>T, p.Arg139Cys) rs147390019 (416G>A, p.Arg139His) rs186364861 (52G>A, p.Val18Ile) rs746071566 (Insertion 36_37insGGAGTC p.Val18_Val19insGlyVal)	Thiopurine intolerance and adverse drug reaction in carriers of the loss-of-function NUDT15 genetic polymorphisms	[41] [42] [43] [36]
*PACSIN2*	Vesicle formation, endocytosis and caveole biogenesis	rs2413739 (T>C)	Severe gastrointestinal and haematological toxicities in TT genotype patients	[70] [71]
*ITPA*	Pyrophosphatase that hydrolyzes the non-canonical purine nucleotides inosine triphosphate	rs1127354 (94C>A, p.Pro32Thr) rs7270101 (IV2 +21A>C)	Severe febrile neutropenia and hepatotoxicity in carriers of the variant alleles	[59] [6]

*A natural substrate for this enzyme has yet to be identified. ITPA: Inosine triphosphate pyrophosphatase; NUDT15: nucleotide triphosphate diphosphatase; PACSIN2: Protein kinase C and casein kinase substrate in neurons protein 2 TPMT: Thiopurine S-methyltransferase

### TPMT

*TPMT* (EC 2.1.1.67) is ~30 kDa cytosolic enzyme expressed ubiquitously in cells. Its function on xenobiotics is very well established: as already mentioned above, it is responsible for the formation of methylated thiopurines derivatives and its activity is inversely related to the cytoplasmic accumulation of TGN^[18]^. In contrast, the endogenous function of *TPMT* is unknown: however, a role in the methylation of endogenous selenoproteins has been hypothesized and a very recent study confirmed *in vitro* a role for *TPMT* in the biosynthesis of selenocysteine^[19]^. Additionally, also a role in the biosynthesis of the molibdenum-binding co-factor molybdopterin has been postulated but not fully demonstrated yet^[20]^. *TPMT* is encoded by a 27 kb gene, composed of 10 exons, located in the short arm of chromosome 6^[21]^. Its expression varies and the patient genetic background is responsible for the observed interindividual differences in clinical efficacy and hematologic toxicity of thiopurine therapy^[22]^. Recent genome-wide association studies (GWAS) have provided evidences of *TPMT* as the only monogenic trait that influences the TPMT phenotype, finding that only variants in *TPMT* gene are significantly associated with TPMT activity in ALL children at genome-wide level (*n* = 1,026; *P* = 8.6 × 10^-61^) and in a mixed-population (two cohorts of adult healthy volunteers and one of pediatric ALL patients, *n* = 1,212; *P* = 1.2 × 10^-72^)^[23,24]^. TPMT activity is inherited as an autosomal codominant trait and approximately 1 in 300 individuals has very low TPMT activity, 10% have intermediate activity and 89% show normal/high activity^[25]^. Indeed 10% of Europeans are positive for a genetic variant in *TPMT* and 0.5% are TPMT completely defective^[26]^. Studies that correlate *TPMT* genotype and phenotype in different populations show that there are more than 38 allelic variants responsible for a possible impairment of the enzyme activity^[27]^. However, only few alleles, such as TPMT*2 and TPMT*3, comprise 95% of the loss of function variants and therefore are clinically relevant. TPMT*2 allele (Single Nucleotide Polymorphism (SNP) rs1800462) is defined by the 238G>C transversion and the p.Ala80Pro amino acid substitution, whereas the TPMT*3 family alleles are defined by the SNPs 460G>A (rs1800460, p.Ala154Thr) and 719A>G (rs1142345, p.Tyr240Cys). In particular, TPMT*3A is the haplotype characterized by both 460G>A and 719A>G transitions, TPMT*3B corresponds to the SNP rs1800460 alone and TPMT*3C to the single SNP rs1142345^[27]^. Genetic variants affecting TPMT activity are different among the main ethnic groups: indeed, the TPMT*3A is the variant most present in Europeans, while TPMT*3C is the most frequent in African and South-east Asian populations (allele frequency of 3%)^[28]^. In Africans, an additional allele putatively presenting reduced enzymatic activity, TPMT*8, identified by the polymorphism rs56161402, is present^[29]^.

All these genetic variations lead to TPMT activity alterations: several studies have shown that the mechanism for decreased levels of TPMT protein and catalytic activity is the enhanced degradation of TPMT allozymes encoded by the TPMT*2 and TPMT*3A alleles^[30-32]^. These TPMT activity alterations are related to a high risk of severe side effects during thiopurine treatment. Many studies have demonstrated that standard thiopurine doses administration is correlated to a higher risk of developing severe and even fatal hematologic toxicity in individuals with low TPMT activity compared to individuals without genetic variations in this gene. In particular, this side effect is due to the accumulation of TGN^[33]^. Murine models knock-out for *Tpmt* do not show any apparent phenotype in the absence of drug challenge, whereas they present differences in TGN concentrations, MMPN concentrations and toxicity among *Tpmt*(-/-), *Tpmt*(+/-), and *Tpmt*(+/+) genotypes when thiopurines are administered, resembling the same phenotype of human individuals carrying *TPMT* defective polymorphisms^[34]^.

Clinical guidelines from the Clinical Pharmacogenetics Implementation Consortium (CPIC) are currently available and recommend to assess *TPMT* status in term of either genetic variations or activity in ALL and IBD patients before starting thiopurine therapy, in order to optimize dosages and thus drug efficacy and tolerance^[35,36]^. Nonetheless, there are many cases of patients without *TPMT* defective variants who are intolerant to a full dose of thiopurines and present leukopenia or other adverse effects, suggesting that also other genes or environmental factors can contribute to the risk of these side effects during thiopurine therapy^[37]^.

### NUDT15

Recent independent agnostic pharmacogenomic studies have identified a novel pharmacogene important for thiopurines: the nudix hydrolase 15 gene (*NUDT15*, also known as *MTH2*), is ~10 kb long, and is located on q region of chromosome 13^[38]^. NUDT15 (EC 3.6.1.9) is an ubiquitously expressed nucleotide triphosphate diphosphatase that converts oxidized GTP to its monophosphate form, preventing the integration of the damaged purine nucleotides into DNA and subsequent mismatch repair^[39]^. The triphosphate thionucleotides are potential substrate for NUDT15 that can hydrolyse and inactivate them. In patients with defective NUDT15, treatment with a standard dose of MP leads to an excessive accumulation of tGTP/tdGTP and consequent extensive DNA damage and cytotoxicity^[40]^.

Yang *et al*.^[38]^ performed an Immunochip-based association study in 978 Korean CD patients treated with thiopurines, investigating the association of 196524 genetic variants previously shown to be genetic susceptibility loci for drug-induced leukopenia in autoimmune or inflammatory diseases. The missense SNP 415C>T of *NUDT15* gene (rs116855232), that induces p.Arg139Cys change, was associated with early leukopenia in the combined analysis of the discovery and replication samples (OR = 35.6; *P* combined = 4.88 × 10^-94^). Similarly, a GWAS study was conducted, using the HumanExomeBeadChip array (244770 SNPs), on a discovery cohort including 657 ALL children and a replication cohort of 371 ALL patients. The association with MP dose intensity (defined as the ratio between clinician-prescribed MP dose and protocol dose) was investigated, finding rs1142345 in *TPMT* and rs116855232 in *NUDT15* as significantly associated loci (*P* ~9 × 10^-9^) with independent replication. Patients that presented a homozygous TT genotype for *NUDT15* rs116855232 were very sensitive to MP and they could tolerate only 8.3% of the usual MP dose; this polymorphism explains 22% of variance in thiopurine-tolerance^[41]^. Moriyama *et al*.^[42]^ reported that *NUDT15* variant p.Arg139Cys did not affect enzymatic activity but rather protein stability, likely due to a loss of supportive intramolecular bonds that caused rapid proteasomal degradation in cells.

Moriyama *et al*.^[47]^ sequenced all exons of *NUDT15* in 3 ALL cohorts including 270 children from Guatemala, Singapore and Japan and identified 4 coding SNPs located in exons 1 and 3 associated with a loss of enzymatic activity (from 74.4% to 100%): the missense SNP rs116855232, the 416G>A SNP (rs147390019) that induces p.Arg139His and two other variants that affect the Val18 residue, the 52G>A resulting in a conversion of valine into isoleucine (p.Val18Ile) and the 36_37insGGAGTC insertion that leads to an in frame addition of a glycine and a valine residue (p.Val18_Val19insGlyVal). They also identified 5 *NUDT15* haplotypes (*1-*5) with distinct combinations of these 4 variants and they classified patients into 3 diplotypic groups: normal activity (*1/*1), intermediate activity (*1/*2, *1/*3, *1/*4 and *1/*5), and low activity (*2/*3, *3/*3 and *3/*5). Loss-of-function *NUDT15* diplotypes were consistently associated with thiopurine intolerance. *In vitro* studies on human lymphoid cell line showed an increased level of active TGTP in the *NUDT15* knock-down cells compared to control cells, with the elevation of intracellular incorporated DNA thionucleotides and consequently higher thiopurines induced apoptosis^[42]^.

A meta-analysis study of *NUDT15* variants investigating 1,752 patients from 7 independent cohorts (both ALL and IBD) showed a significant association between *NUDT15* rs116855232 T allele and myelotoxicity: indeed, the presence of the T allele increases ~8 times the probability to have leukopenia in comparison to the C allele (*P* < 0.00001, OR = 7.86, 95% CI: 6.13, 10.08). Analysing 2745 patients from 13 cohorts, the authors also found that T carriers tolerated lower mean daily thiopurines dose (*P* < 0.00001)^[43]^. A second meta-analysis, including 16 studies on rs116855232 (p.Arg139Cys), confirmed its role as clinically relevant predictor of thiopurine-induced leukopenia^[44]^. This SNP is the most common *NUDT15* genetic variant, particularly frequent in East Asians (10.4%) and Hispanics (7.1%), but almost absent in Europeans (0.46%) and Africans^[45]^. Distribution of other *NUDT15* genetic variants are also ethnic specific: rs147390019 is common only in Hispanics (1.7%)^[43]^ and rs186364861 in Asians (1.6%)^[43]^. Recently, a *NUDT15* variant with a significant prevalence in Caucasian patients was discovered in a cohort of patients with IBD, rs746071566, (p.Gly17_Val18del) and was associated with myelosuppression during thiopurine therapy^[46]^. The relevance of this variant in Caucasian patients with ALL has still to be fully understood.

International guidelines of thiopurines treatment suggest to consider *NUDT15* together with *TPMT* genotypes before drug administration. Patients who present the above-mentioned genetic variants need to adjust drug dosage. CPIC suggests to use the normal starting dose for *NUDT15* normal metabolizers (e.g., *NUDT15* *1/*1, MP 75 mg/m^2^/day in ALL) and a reduced one in intermediate metabolizers (e.g., *NUDT15* *1/*3, MP 30%-80% normal starting dose) and poor metabolizers (e.g., *NUDT15* *3/*3; MP 10 mg/m^2^/day in ALL)^[36]^.

Moriyama *et al*.^[47]^ evaluated the effects of *NUDT15* on thiopurines metabolism and identified DNA-incorporated thioguanine (DNA-TG) and TGN as pharmacologic markers for NUDT15 genotype-guided thiopurines dosing. They analyzed the level of these metabolites in a cohort of 55 Japanese ALL pediatric patients: those with *NUDT15* deficiencies accumulated DNA-TG more efficiently than patients without *NUDT15* variants (*P* = 4.0 × 10^-9^). They also showed that cytosolic TGN and nuclear DNA-TG were positively correlated with each other (*P* = 6.5 × 10^-4^), but the ratio of DNA-TG to TGN was significantly higher in *NUDT15* deficient patients (*P* = 3.6 × 10^-9^), suggesting that DNA-TG is a more relevant MP metabolite than TGN to inform *NUDT15* genotype-guided dose adjustments^[47]^.

### ITPA

Human inosine triphosphate pyrophosphatase (ITPA, EC 3.6.1.19) is a ~21 kDa enzyme that is ubiquitously expressed in the cytoplasm of cells and acts in homodimer form^[48]^. ITPA catalyses the hydrolysis of the triphosphate moieties from noncanonical (deoxy-) purine triphosphate, such as inosine triphosphate, deoxy-inosine triphosphate, xanthosine triphosphate, deoxy-xantosine triphosphate (ITP, dITP, XTP and dXTP respectively), thus recovering the monophosphate derivatives and releasing the pyrophosphate group. IMP/dIMP are fundamental intermediates in purine metabolism and can be converted into canonical adenosine monophosphate (AMP/dAMP, and therefore ATP/dATP) and guanosine monophosphate (GMP/dGMP, hence GTP/dGTP). It has been estimated that nearly one third of the human population has a genetically determined decreased ITPase activity^[49]^. The resulting unusual accumulation of noncanonical purine triphosphate can be toxic for the cells because of their interference with polymerase enzymes, and their incorporation into DNA or RNA. The 19 kb *ITPA* gene is located on the short arm of chromosome 20 and includes 8 exons^[50]^. Genetic knock-out (Itpa-/-) mice present abnormal features of growth retardation and cardiac myofiber disarray and die within 2 weeks after birth, likely because of the accumulation of ITP in the nucleotide pool found in erythrocytes^[51]^. Some reports have associated *ITPA* variants to human diseases, such as psychiatric disorders, encephalopathy, young-onset tuberculosis and infertility^[49,52-54]^. The human *ITPA* gene carries several polymorphisms associated with a reduction of enzymatic activity among which *ITPA* SNPs rs1127354 and rs7270101. SNP rs1127354 consists in the C94A transversion at the exon 2 level, leading to the amino acid substitution p.Pro32Thr. This substitution determines complete depletion of ITPA activity in homozygous individuals and a reduction to about 25% in heterozygous individuals^[55]^. The frequency of this polymorphism is around 5%-7% in the Caucasian population, but varies greatly depending on the ethnicity ranging from 2% in Hispanic populations up to 19% in Asians^[56]^. rs7270101 instead is the intronic transversion IVS2 +21A>C, that alters a very conserved adenine at the level of a splicing site, thus leading to an aberrant mRNA. This variant has a less severe effect than the C94A alteration: indeed, patients heterozygous for this SNP show an activity of about 60% compared to wild-type^[55]^. Genetic variants in *ITPA* are of major interest in pharmacogenetic research being associated with altered outcomes for patients undergoing antimetabolite therapy, in particular antileukemic agents such as thiopurines and methotrexate or antivirals such ribavirin or zidovudine^[57,58]^.

The occurrence of toxicities during the ALL maintenance therapy was previously associated with *ITPA* variants: in the context of the SJCRH Total XIIIB protocol, Stocco *et al.*^[59]^ found that ALL patients with a variant *ITPA* allele had a higher probability of developing severe febrile neutropenia. Increased accumulation of MMPN nucleotides has been described both in patients’ blasts, during a mercaptopurine-based window therapy, and erythrocytes, during maintenance therapy with MP doses adjusted for *TPMT* genotype. Adam de Beaumais *et al*.^[6]^ found that wild-type *TPMT*/variant *ITPA* ALL patients treated with the EORTC (European Organization for Research and Treatment of Cancer)-58951 protocol had higher MMPN concentration in erythrocytes in comparison to wild-type *TPMT*/wild-type *ITPA* and that a MMPN threshold above 5000 pmol/8 × 10^8^ RBC was associated to an increased risk of hepatotoxicity. Controversial results were reported in Asian populations: Kim *et al*.^[60]^ did not find a difference in the cumulative incidence of grade III/IV febrile neutropenia according to *ITPA* genotypes in Korean ALL pediatric patients whereas Malaysian patients with *ITPA* 94A allele seemed more prone to develop fever and liver toxicity in therapeutic protocols not individualized for *TPMT*^[61]^. An Italian pharmacogenetic multicentric study was performed on 508 pediatric ALL patients treated with the AIEOP-BFM 2000 protocol: four children were homozygous variant for rs1127354 (94 AA genotype) and had a significant 13-fold increase in the risk of developing severe (grade III-IV) neurological toxicities during the 2 months induction phase, when compared with wild-type subjects, and showed also an higher risk of developing severe gastrointestinal complications (8-fold increase in risk)^[62]^. However, in this study, MP were introduced only four weeks after the beginning of the treatment, thus the incidence of these adverse effects could not be uniquely ascribable to thiopurines and the contribution of other drugs, such methotrexate, could be considered.

### PACSIN2

Protein kinase C and casein kinase substrate in neurons protein 2 (PACSIN2) is a member of the PACSIN protein family^[63]^. PACSIN2 is an ubiquitously expressed protein of ~56 kDa and is localized in physical proximity to membranes. Structurally, PACSIN2 contains a membrane-sculpting Bin-Amphiphysin-Rvs (BAR) domain at N-terminal, a central region with a proline rich motif and a C-terminal Src homology 3 (SH3) domain^[64-65]^. The F-BAR domain regulates membrane curvatures and invaginations formation involved in vesicle budding, endocytosis and caveole biogenesis^[66-68]^, whereas the SH3 domain mediates the interaction with many membrane proteins. PACSIN2 is encoded by a 180kbp gene located in the long arm of chromosome 22 which includes 15 exons. Human and murine *PACSIN2* cDNA and protein show significant homology with 79.8% and 93.6% identity, respectively. The *PACSIN2* knock-out mouse presents a decreased mean corpuscular volume, decreased mean corpuscular hemoglobin concentration, decreased leukocyte cell number, increased heart weight and improved glucose tolerance^[69]^. A recent genome wide analysis on a panel of 30 human HapMap cell lines trios demonstrated that the intronic SNP rs2413739 (T>C) of *PACSIN2* can influence TPMT activity. In ALL patients enrolled in the Total XIIIB/XV protocols at SJCRH, the TT genotype was associated with lower TPMT activity in patients’ RBC and with an higher rate of gastrointestinal toxicity during thiopurines therapy. This latter association was also validated in an independent cohort of ALL patients treated with the AIEOP-BFM ALL 2000 protocol^[70]^. A retrospective study in pediatric ALL patients treated with thiopurines suggested that the presence of rs2413739 in *PACSIN2* gene is a significant risk factor for mercaptopurine induced hematological toxicity in patients with wild-type *TPMT*^[71]^. It could be hypothesized that the increased toxicities observed in rs2413739 TT carriers are due to lower TPMT activity. However, no contribution of rs2413739 on thiopurine metabolites (TGN and MMPN) was reported, thus the mechanistic link between the *PACSIN2* SNP and thiopurine adverse effects is not yet clearly established.

The PACSIN2 SH3 domain can interact with other proteins, such as Ras-related C3 botulinum toxin substrate 1 (Rac-1)^[67]^. Several evidences suggest a possible interplay among PACSIN2, Rac-1 and thiopurines. The physical interaction between the proline-rich hypervariable region of Rac-1 and the SH3 domain of PACSIN2 is well known. Due to its role in intracellular vesicle-mediated transport, PACSIN2 could serve as a molecular brake on Rac-1 signaling, by mediating the Rac-1-GTP internalization toward early endosomes, thus slowing down cell processes, such as growth and differentiation, regulated by the small GTPase^[72]^. The thiopurine metabolite tGTP binds to Rac-1 as a competitive antagonist of GTP: this binding suppresses the activation of Rac-1 and leads to apoptosis. Indeed, in IBD patients, effective MP therapy led to decreasing concentrations of Rac-1-GTP and Rac-1 expression^[73]^. PACSIN2 moreover may affect TPMT activity: indeed, the knock-down of PACSIN2 mRNA in human leukemia cells NALM6 resulted in significantly lower TPMT expression and enzymatic activity^[70]^ that can in turn result in a higher tGTP availability and Rac-1 inhibition. Although not clear enough, these evidences suggest that PACSIN2, Rac-1 and their expression level could be additional potential biomarkers of thiopurines response and should be further investigated.

### Rac-1

Rac proteins play a major role in T cell development, differentiation, and proliferation^[74]^ and Rac-1 knock-outs are embryonic lethal in mice^[75]^. Rho GTPase proteins cycle between a GDP-bound and a GTP-bound state through the action of guanine nucleotide exchange factors (GEFs) and the opposing GTPase activating proteins (GAP); the GTP-bound state is generally considered as the active conformation, regulating signaling pathways in cells. The binding of tGTP instead of GTP on Rac-1 suppresses its activation, and thus the activation of its downstream targets such as mitogen-activated protein kinase, NF-κB and the antiapoptotic protein Bcl-xL. *In vivo* and *in vitro* studies suggested that Rac-1 plays a key role in thiopurines mechanism of action^[24]^. Tiede and collaborators have demonstrated that binding of AZA-generated tGTP to Rac-1 induced the mitochondrial pathway of apoptosis in primary CD4+ T lymphocytes lamina propria cells of healthy volunteers and CD patients, by downregulating Bcl-xL mRNA and protein^[24]^. An *in vitro* study on primary T cells isolated from healthy donors clarified the molecular mechanism beneath the tGTP-block of Rac-1, showing that the accumulation of inactive tGDP-Rac-1 proteins over time depends on the inhibition of the GEF factor Vav^[76]^. IBD patients treated with these drugs present a reduced median Rac-1 expression compared with patients without immunosuppressive therapy, and among MP treated patients, non-responders showed an increased median active Rac-1 expression^[73]^. In animal models, neutrophil and macrophage conditional *Rac1* knock-out show normal bowel histology and do not present any obvious colonic phenotype; nonetheless they are protected against dextran sulphate sodium-induced colitis and show a reduced level of the proinflammatory cytokine IL-1β and of the neutrophil chemokine KC compared to wild-type mice^[77]^. The pharmacogenetics of *RAC1*, a ~30 kDa gene located on p arm of chromosome 7, has been investigated in IBD, but not in ALL patients. Two intronic *RAC1* polymorphisms (rs10951982 and rs4720672) have been associated with UC susceptibility in a discovery and in 2 independent replication cohorts; IBD patients who had the rs10951982 G risk allele had increased expression of *RAC1* compared to those without this allele^[77]^. The same SNPs, together with *RAC1* SNP rs34932801, was genotyped in 59 IBD pediatric patients and no association was found with thiopurine effectiveness after 12 months of therapy^[78]^. In contrast, a retrospective report on a cohort of 156 thiopurine-treated CD adults has shown an association between the GC heterozygous genotype in SNP rs34932801 and poorer response to thiopurines, in comparison to wild-type GG^[79]^.

### Epigenetics

Epigenetic factors, in particular considering molecular factors that can be inherited from one generation to the other but that do not depend on the DNA sequence, such as methylation of cytosines on DNA and non-coding RNA profiles, such as microRNAs (miRNAs) and long non-coding RNAs, have been recently considered as innovative pharmacogenomic determinants of interindividual variability in drug efficacy and toxicity^[80]^.

For thiopurines, studies on the effect of pharmacoepigenetic factors are still limited. Thiopurines have been shown to reduce global DNA methylation^[81]^. This effect was demonstrated *in vitro* in human leukemia cells and has been related to the reduction of *de novo* purine synthesis (DNPS) induced by thionucleotides, which determines reduction in ATP levels and depletion of S-adenosylmethionine (SAM) concentration, an important co-factor of methyltransferases, including DNA methyltransferases, enzymes involved in the regulation of DNA methylation^[82]^. Also TPMT activity, by modulating the capability of thionucleotides to inhibit DNPS, has been shown to influence the reduction of DNA methylation induced by mercaptopurine and thioguanine^[83]^. However, how this effect of thiopurines on DNA methylation may be related to interindividual variability in efficacy and adverse effects of these drugs or on the expression of candidate genes involved in thiopurines pharmacokinetics and pharmacodynamics is still unknown.

In the treatment of pediatric ALL, mercaptopurine is generally associated with methotrexate, both during consolidation and maintenance therapies. The epigenetic factors influencing interindividual variability in response to these phases of therapy may be of relevance also for thiopurine effects. For examples, a major adverse effect of consolidation therapy for pediatric ALL is oral mucositis, which is generally ascribed to methotrexate. However, as already mentioned, in some protocols, such as SJCRH Total XIIIB protocol, this adverse effect has been related also to TPMT activity^[70]^, which indicated that also interindividual variability in mercaptopurine disposition may influence this adverse effect. A recent study evaluated DNA methylation profiles associated with oral mucositis during consolidation therapy in children with ALL. This study confirmed also in patients a significant reduction in SAM levels during methotrexate/mercaptopurine therapy (doses respectively 5 g/m^2^ every 2 weeks with leucovorin rescue 15 mg/m^2^/dose and daily mercaptopurine 25 mg/m^2^). In this study, DNA methylation was measured considering the percentage of 12 CpG islands in the LINE1 gene, that is considered a surrogate for DNA global methylation^[84]^. Intriguingly, a small increase in this DNA methylation marker was observed during therapy, with no correlation with SAM levels and incidence of oral mucositis. One limitation of this study is that DNA was extracted from peripheral blood mononuclear cells and since DNA methylation is cell specific, different effects could be observed in tissues more directly involved in methotrexate/mercaptopurine, effects such as residual lymphoblasts and epithelial cells of the gastrointestinal tract.

Considering non coding RNAs, studies are also limited, in particular for the association with the clinical effects of thiopurines. One study considered miRNA profiles after treatment with mercaptopurine in rat placenta, as a model to study toxicity of this drug in fetal development. Differentially expressed miRNAs could be identified and were involved in process such as apoptosis, inflammation and ischemia^[85]^.

Non coding RNAs affecting the expression of pharmacogenes in human liver have been identified and associated with age. Among these genes, also those relevant for thiopurines pharmacokinetics have been considered^[86]^. Results showed that some miRNAs were associated with both age and *TPMT* expression; in particular, hsa-miR-34a-5p and has-miR-125b-5p showed the largest significant negative correlation with *TPMT* expression. Interestingly, TPMT activity measured in erythrocytes is negatively associated with age in children with ALL^[23]^ and age has been shown to modulate thiopurine pharmacokinetics in pediatric patients^[87]^. The molecular mechanisms by which age influences TPMT activity could involve epigenetic factors, however more research is needed to clarify this phenomenon.

While the contribution of epigenetic factors to mercaptopurine induced adverse effects in pediatric ALL is still uncertain and speculative, further studies on this topic may bring important insights to improve therapy of patients taking mercaptopurine.

### Genotyping *vs.* phenotyping

Many genes involved in thiopurine pharmacogenetics (i.e., *TPMT, NUDT15, ITPA*) encode for enzymes whose activity can be measured in patients, for examples in erythrocytes. For *TPMT* there are many studies considering both phenotyping and genotyping, while for the other enzymes the information available is still limited.

Both *TPMT* genotyping or phenotyping before starting thiopurines treatment allows to identify patients with reduced enzymatic activity and increased risk of drug induced adverse effects and to adjust starting dose accordingly or to use an alternative therapy. There is a risk of *TPMT* misclassification when only genotyping or phenotyping is used. Genotype-phenotype concordances are usually set between 76% and 99%^[22]^. Indeed, the conduction of both tests may give clinicians a more complete picture about patient’s risks during therapy, but there is no consensus on the evaluation of both in all patients, also for the associated costs. The Food and Drug Administration (FDA) still indicates genotyping or phenotyping before drug administration to identify *TPMT* status^[88]^, while the American College of Gastroenterology indicates phenotyping as the first choice for patients using thiopurines for IBD because phenotyping reports a quantitative level of TPMT enzyme activity^[89]^. Genotyping certainly detects variations useful to predict the enzyme activity and the possible development of adverse effects directly associated with enzyme deficiency. One advantage of genetic tests is that they can be performed before starting therapy and the information obtained will be unchanged for the entire patient lifespan. Despite the relatively simple and rapid procedure, genetic tests provide data about key variants generally detecting the most common genetic variants associated with enzyme decrease but is not able to give a full prediction because they do not verify the presence of rare or previously undiscovered variants that will not be detected by variant-specific genotyping methods^[90]^. The advent of genome wide and next-generation sequencing (NGS) approaches and the continued reduction in the costs of these technologies will partly overcome this problem. Moreover, the genotype is not solely responsible for patient’s susceptibility of a drug. Genotyping tests will always provide a partial point of view since they do not consider variability of enzyme activity due to the presence of drug interactions, presence of others pathologies or other epigenetic factors. Phenotyping consists in a more direct observation of how the drug is metabolized and considers also inter-individual differences between patients with the same genetic arrangement. For this purpose, the activity of TPMT in lysed erythrocytes can be directly and successfully determined using reversed-phase high performance liquid chromatography (HPLC)^[91]^. It is important to measure TPMT activity on a blood sample taken before starting thiopurine administration and during therapy, because its activity can increase during treatment and thus can interfere with test results^[36]^. Moreover, phenotyping test shall be performed at least 3 months after a transfusion of erythrocytes^[92]^: during ALL chemotherapy many patients receive transfusions therefore the utility of phenotyping in this setting may be limited.

In conclusion, *TPMT* genotyping and phenotyping provide complementary information. Despite the usually quite high levels of concordance between the analysis, the combination of both genotype and phenotype tests insures a higher precision in the treatment personalization.

## Conclusion

With the development of highly efficient sequencing techniques and the expected more diffuse availability of patient specific genotyping data, particularly for severe life threatening disease such as pediatric ALL, thiopurines pharmacogenetics may significantly contribute to reduce adverse effects and improve efficacy, by considering multilocus genotyping of *TPMT* and *NUDT15*. More research is needed to further improve pharmacogenetics of thiopurines by including additional gene variants, such as *ITPA* and *PACSIN2*, to obtain further clinical and mechanistic evidence.

## Declarations

### Copyright

© The Author(s) 2019.
